# Process evaluation of the MOSAIC trial: treatment experience of two psychological therapies for out-patient treatment of Anorexia Nervosa

**DOI:** 10.1186/s40337-016-0091-5

**Published:** 2016-02-09

**Authors:** Kelly Ann Zainal, Beth Renwick, Alexandra Keyes, Anna Lose, Martha Kenyon, Hannah DeJong, Hannah Broadbent, Lucy Serpell, Lorna Richards, Eric Johnson-Sabine, Nicky Boughton, Linette Whitehead, Janet Treasure, Ulrike Schmidt, on behalf of the MOSAIC trial group

**Affiliations:** Section of Eating Disorders, Department of Psychological Medicine, Institute of Psychiatry, Psychology and Neuroscience, King’s College London, PO Box 59, SE5 8AF London, UK; Hope Wing, Porters Avenue Health Centre, North East London Foundation Trust, Dagenham, Essex UK; Eating Disorders Research Group, Research Dept. of Clinical, Educational & Health Psychology, University College London, London, UK; The Phoenix Wing, St Ann’s Hospital, Barnett, Enfield and Haringey Mental Health Trust, Tottenham, London, UK; Oxford Adult Eating Disorder Service, Cotswold House, Warneford Hospital, Oxford NHS Foundation Trust, Oxford, UK

**Keywords:** Anorexia nervosa, MANTRA, Randomised–controlled trial, Specialist supportive clinical management, Process evaluation

## Abstract

**Background:**

This study is part of a series of process evaluations within the MOSAIC Trial (Maudsley Outpatient Study of Treatments for Anorexia Nervosa and Related Conditions). This randomised controlled trial (RCT) compared two psychological treatments, the Maudsley Model for Treatment of Adults with Anorexia Nervosa (MANTRA) and Specialist Supportive Clinical Management (SSCM) for adult outpatients with Anorexia Nervosa. The present process study integrates quantitative (treatment acceptability and credibility) and qualitative (written) feedback to evaluate patients’ treatment experiences.

**Method:**

All 142 MOSAIC participants were asked to (a) rate treatment acceptability and credibility on visual analogue scales (VAS) at six and 12 months post-randomisation, and (b) provide written feedback regarding their views on their treatment at 12 months. Transcripts were first analysed thematically and then rated according to the global valence of feedback (positive, mixed/negative).

**Results:**

114/142 (80.3 %) MOSAIC participants provided VAS data and 82 (57.7 %) provided written feedback. At 12 months, MANTRA patients gave significantly higher acceptability and credibility ratings compared to SSCM patients. A significantly higher proportion of MANTRA patients provided written feedback. MANTRA patients also tended to write in more detail and to give globally more positive feedback when compared to individuals receiving SSCM. Qualitative themes suggest that patients experienced the two treatments differently in terms of characteristics and outcomes.

**Conclusions:**

This study highlights the benefits of incorporating qualitative and quantitative data into RCT process evaluations. MANTRA patients were more willing to express their views on treatment and generally felt more positively about this than those receiving SSCM.

## Background

Anorexia nervosa (AN) is a highly debilitating mental disorder which is challenging to treat and expensive to manage, especially in adults with a more enduring form of the illness [1]. The UK National Institute for Health and Care Excellence [2] and the US National Institute of Mental Health [3] have emphasized the need for the development and evaluation of new approaches to the treatment of AN as at present, there is little high quality empirical evidence to support a preferred psychological treatment [4].

Whilst randomised controlled trials (RCTs) remain the gold standard for evaluating treatment outcomes, process evaluations integrated into trials provide crucial information regarding factors involved in treatment success, such as fidelity and quality of implementation, potential causal mechanisms, and contextual factors associated with variation in outcomes [5]. Such process evaluations are used to supplement quantitative outcomes by providing a more complete picture of the treatment approach [6]. This puts an emphasis on meanings, experiences, and views of all the participants in order to understand treatment in the natural setting [7]. Evaluations aim to identify the potential “active ingredients” of a particular treatment approach, and explore interactions between patients and therapists [6].

The present study is the second stage of a planned process evaluation conducted as part of a large multi-centre RCT, the Maudsley Outpatient Study of Treatment for Adults with Anorexia Nervosa and Related Conditions (MOSAIC). The MOSAIC trial compared two outpatient treatments. The first is a novel therapy, Maudsley Model of Treatment for Adults with Anorexia Nervosa (MANTRA) [8–10], and the second, Specialist Supportive Clinical Management (SSCM) is a more established treatment that was developed as a standardised form of treatment as usual, emphasising the importance of the non-specific curative factors of therapeutic relationships and active interest of the therapists in the progress of their patients. This treatment has been shown to be superior to cognitive behavioral therapy and interpersonal psychotherapy at end of treatment [11], but with effects lessening over time [12, 13]. Full details on the MOSAIC trial [14], MANTRA [8–10, 15, 16] and SSCM [11, 17] can be found elsewhere. Both treatments were found to be effective, with MANTRA having advantages in more severely ill patients [18].

In the first stage of our process evaluation, we explored both therapist and patient experiences of MANTRA and SSCM using in-depth qualitative interviews [19, 20]. There was a high level of agreement between therapists’ and patients’ views of these two treatments, their unique and overlapping features and relative strengths and weaknesses.

A limitation of the study on patients’ views [19] was that because of resource constraints only a small proportion (*n* = 17) of participants from one of the study centres were included and they were interviewed at different stages of treatment, i.e. without the benefit of having experienced a full course of treatment. Oakley et al. [21] have highlighted several methodological issues to be considered in conducting process evaluations within RCTs: All participating treatment sites should be included as well as both qualitative and quantitative data, and importantly, process data should be analysed *prior* to the outcome data to minimize potential biases in interpreting results. The present study has taken into account the aforementioned criteria, in addition to approaching all available MOSAIC trial patients to obtain generalisable results and assessing their feedback after the completion of all treatment sessions, in order to supplement the earlier study by Lose et al. [19].

Accordingly, the aim of the current study was to conduct a process evaluation prior to analysis of outcome data to investigate patients’ experiences of two different treatments within a large RCT of outpatient treatment of AN. The main objective was to combine participants’ quantitative treatment acceptability and credibility ratings at 12 months with qualitative written feedback. It was hoped that the patients’ qualitative feedback could shed more light on the factors associated with their treatment ratings.

## Materials and Method

### Participants

MOSAIC trial participants were 142 adults (98 % female) with a DSM-IV-TR (2000) diagnosis of AN or Eating Disorder Not Otherwise Specified AN type (EDNOS-AN), recruited between June 2010 and November 2012 from four treatment centres in the UK: South London and Maudsley NHS Foundation Trust; North East London Foundation Trust Eating Disorders Service; Barnet, Enfield & Haringey Mental Health NHS Trust; Oxford Health NHS Foundation Trust. For inclusion in the trial, participants needed a body mass index (BMI) of below 18.5 kg/m^2^ and the absence of any major mental or physical co-morbidities that warranted their own treatment. There was no lower BMI limit provided patients were medically stable. After initial assessment, including a range of eating disorder (ED) and other outcome measures, participants were randomly allocated to receive either 20 once-weekly sessions of MANTRA or SSCM plus four monthly follow-up sessions. Optional additional sessions with a dietician and sessions including a family member were also offered. Patients with a BMI of ≤ 15 kg/m^2^ at the start of treatment were offered 30 weekly sessions. Therapists were expected to see patients in both treatment conditions to control for therapist effects. Follow-up research assessments were carried out at six months (approximately at the end of weekly treatment sessions) and 12 months (end of follow-up sessions). Full details of in- and exclusion criteria and trial procedures can be found elsewhere [14].

Informed consent was obtained from all individual participants included in the study. Ethical approval for the MOSAIC trial was obtained from the Central London REC 4, National Research Ethics Service, Royal Free Hospital, London, (Reference: 10/H0714/9). The trial is registered with Current Controlled Trials: ISRCTN67720902.

### Measures

#### Eating Disorders Examination (EDE) [22]

The EDE measures eating disordered thoughts and behaviors. The EDE is a semi-structured diagnostic interview that generates 4 subscale scores, the mean of which gives an overall global score. Higher scores indicate more serious eating disordered psychopathology. It has been shown to have good reliability and discriminant validity [23, 24]. EDE interviews were carried out by trained assessors. Inter-rater reliability was checked through second scoring every 10^th^ interview.

#### Treatment acceptability and credibility

Acceptability and credibility of treatment were assessed using two 10 cm visual analogue scales (VAS). Participants were asked to place a vertical mark on each scale to indicate how they would rate themselves in relation to two questions: 1) ‘How *acceptable* did you find the type of treatment you received from your therapist during this study?’ and 2) ‘To what extent do you feel that the treatment you received has *helped you* to reduce your eating disorder behaviors?’ On the two scales, 0 cm corresponds to ‘completely unacceptable’ and ‘not at all’ and 10 cm represents ‘completely acceptable’ and ‘very much so’ respectively. As a treatment’s ‘credibility’ may be difficult for patients to conceptualize, we operationalised it as the reduction of eating disorder behaviours to assess how the treatment has been effective. Helpfulness of treatment is a key aspect of established credibility scales [25]. Scores for treatment acceptability and treatment credibility were calculated separately and to one decimal place.

#### Participant written feedback form

This qualitative feedback form asked two questions: (1) ‘What was your experience of therapy in the MOSAIC trial?’ and (2) ‘Do you have any comments or suggestions?’ Participants were informed that anonymised quotes might be used in publications and a tick box was provided if participants wished to opt out from this (2/82 participants used this option). To protect anonymity, all participants who completed the written feedback were assigned a pseudonym.

### Procedure

Demographic and clinical information including age, age of onset, illness duration, BMI, and EDE global score were collected at baseline (pre-randomisation) assessment. Process evaluation data were part of the planned 6- and 12 month follow-up assessments. Ratings of acceptability and credibility were obtained at both 6- and 12-month follow-up assessments in order to capture changing views over time. Written feedback was collected at 12 months only. In all cases, participants were posted the process questionnaires to complete in their own time prior to attending the planned follow-up appointment.

At the time of data collection, research assessors did not read written feedback forms in order to ensure they remained blind to patient treatment allocation. Instead a separate researcher (KAZ), who was not involved in data collection, read and transcribed the feedback for analysis.

### Data analysis

#### Quantitative analysis

Analyses were conducted with IBM SPSS Statistics Version 20 software. Baseline demographic and clinical characteristics were compared between the two treatment groups and between patients who did or did not provide process evaluation data. Independent *t*-tests were conducted to compare the acceptability and credibility ratings given by MANTRA and SSCM patients at 6-month and12-month follow up assessments.

#### Qualitative analysis

Participants’ written feedback forms were transcribed verbatim and imported into NVivo 10 by researcher KAZ. Data were analysed using Braun and Clarke’s [26] six phase thematic analysis process from a realist epistemological standpoint. The approach used is similar to that of our first published process evaluation of the MOSAIC trial [19, 20], in which coding was driven by the information obtained from the participants’ feedback.

All 82 written feedback responses were transcribed into the software package, before being read and re-read to familiarize the researcher with the data and allow for initial generation of ideas. Through repeated re-reading of the transcripts the researcher identified patterns of common issues raised and data were systematically coded (i.e. a code representing the most basic segment or element of the raw data). At this early stage, as many codes as possible were generated and collated, then grouped into overarching themes and subthemes. Subsequently, codes and themes were constantly reviewed and refined, discarded, combined or split. Broad, higher-order themes and subthemes were labeled and organized to include all relevant codes.

**Fig. 1:**
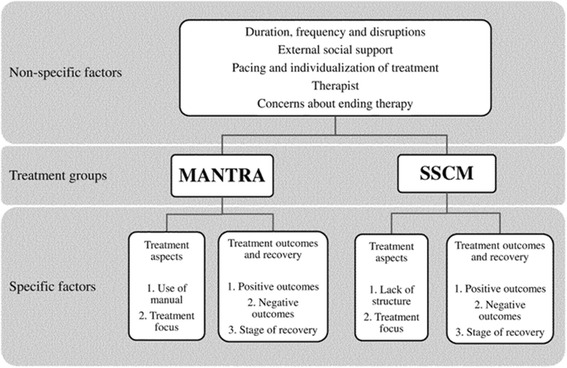
Thematic map of participants’ written feedback

KAZ, BR and US, researchers blind to treatment outcome, completed data triangulations to improve validity and reliability of the resulting themes, noting down over-arching themes and additional ideas. They came together to consolidate their findings, discussing any disagreements until a consensus was reached. The frequencies with which the codes and themes occurred were calculated and the numbers of words in the transcripts were counted, using the word count facility in Microsoft Office Word 2007. The final thematic map is presented in Fig. 1.

#### Global valence ratings of feedback

In a separate step, two researchers (KAZ, BR) independently rated the overall global valence of participants’ responses on the feedback form as overall positive, mixed or overall negative. Feedback was rated as overall positive if the experience of therapy was described using only favorable, approving, enthusiastic descriptions (e.g. *“BRILLIANT… Really feel that I have been so lucky to be here and it has made all the difference between recovery and not”*), as mixed if there clearly were some positive and some negative descriptions (e.g. *“Mainly positive – although I don’t think it was very positive or useful for the state I was in at the time so didn’t make best use of the trial”*), and as overall negative if the person described their experience in dismissive, disapproving or unenthusiastic terms (e.g. *“Useless and made me worse until landed up in hospital again”*). Seven irrelevant responses were excluded (e.g. where participants had exclusively focused their feedback on the research process or were unable to give appropriate feedback on treatment due to receiving a very limited number of sessions). The ratings were finalised when a consensus was agreed upon. These ratings were subsequently used as comparisons between treatment groups in our quantitative analyses.

## Results

### Quantitative analyses

114 of 142 MOSAIC participants (80.3 %) provided acceptability or credibility ratings at six- or 12-month follow-up. There were no baseline differences in age, age of onset, illness duration, BMI or EDE global score between those who did (*n* =114) or did not (*n* =28) provide any feedback at 6-month or12 month follow-up. In addition, 82 of the 142 MOSAIC participants (57.7 %) also provided written feedback. Participants who did or did not provide written feedback did not significantly differ from each other on baseline demographic or clinical characteristics.

A total of 60 MANTRA and 54 SSCM patients provided either quantitative and/or qualitative views on their treatment at 6- or12-month follow-up. MANTRA and SSCM patients who provided any feedback did not differ significantly from each other on baseline demographic or clinical characteristics (see Table 1). A significantly higher proportion of MANTRA patients (48/60, 80 %) provided written feedback compared to those receiving SSCM (34/54, 62.9 %) (Pearson’s Chi-Square 4.028; *df* =1; *p* = 0.045).

**Table 1:** Baseline demographic and clinical characteristics of process evaluation participants split by treatment group

	MANTRA	SSCM	*t*	*df*	*p*
	*n* = 60	*n* = 54			
Age (years), mean (*SD*)	27.0 (8.1)	25.9 (7.6)	.779	112	.438
Age of onset (years), mean (*SD*)	17.7 (6.8)	18.6 (7.0)	-.710	106	.480
Illness duration (years), mean (*SD*)	9.3 (8.2)	7.4 (6.8)	1.351	108	.179
EDE global score, mean (*SD*)	3.2 (1.3)	3.4 (1.3)	-.674	112	.502
BMI (kg/m^2^) mean (*SD*)	16.7 (1.2)	16.7 (1.3)	-.048	111	.961

*Note.* MANTRA: The Maudsley Model of Anorexia Nervosa Treatment for Adults, SSCM: Specialist Supportive Clinical Management, EDE: Eating Disorders Examination, BMI: Body Mass Index

Whilst at six months there was no significant difference on acceptability and credibility between the two treatments, at 12 months. MANTRA patients rated their treatment as significantly more acceptable and credible than SSCM patients (see Table 2).

**Table 2:** Treatment acceptability and treatment credibility ratings across six- and 12- month follow up split by treatment group

6-month follow up					
	MANTRA	SSCM	*t*	*df*	*p*
	*n* = 55	*n* = 47			
Treatment acceptability, mean (*SD*)	8.5 (2.0)	8.0 (2.2)	1.331	100	.186
Treatment credibility, mean (*SD*)	6.4 (3.1)	5.8 (2.7)	1.052	100	.295
12-month follow up					
	MANTRA	SSCM	*t*	*df*	*p*
	*n* = 50	*n* = 43			
Treatment acceptability, mean (*SD*)	8.6 (1.8)	7.8 (2.3)	2.010	91	.047*
Treatment credibility, mean (*SD*)	6.8 (3.1)	5.5 (2.7)	2.244	91	.027*

*Note.* *p < .05. MANTRA: The Maudsley Model of Anorexia Nervosa Treatment for Adults, SSCM: Specialist Supportive Clinical Management, EDE: Eating Disorder Examination, BMI: Body Mass Index

In the written feedback, the mean number of words used was higher in the MANTRA group (*M =* 107.8, *SD =* 72.6) compared with the SSCM group (*M =* 90.6, *SD =* 47.3) although this difference was not statistically significant (*t* =1.296; *df* = 79.9; *p* = 0.199). After excluding participants who had given an irrelevant written feedback response [MANTRA: 4/48 (8.3 %); SSCM: 3/34 (8.9 %)], assessment of global valence showed that in the MANTRA group, 35/44 participants (79.5 %) were positive about their treatment and 9/44 (20.5 %) either had mixed views or felt negatively about treatment, whereas in the SSCM group, 19/31 (61.3 %) were positive and 12/31 (38.7 %) mixed or negative. Differences between the two treatments were not significant (Pearson’s Chi-square value = 2.377; *p* = 0.123).

In both treatment groups, those who provided positive feedback also gave significantly higher acceptability and credibility ratings at 12 months than those who gave mixed or negative feedback. In the MANTRA group, mean acceptability ratings for participants giving positive feedback were: *M* = 9.4, *SD* = 0.9 and for those giving mixed or negative feedback were: *M* = 5.6, *SD* = 1.6, (*t*(41) = 9.725, *p* < .01). Respective numbers for credibility ratings were positive: *M* = 8.1, *SD* = 2.1; mixed or negative: *M* = 3.0, *SD* = 1.6, (*t*(41) = 6.956, *p* < .01). In SSCM, mean acceptability ratings for people giving positive feedback were: *M* = 8.6, *SD* = 1.5 and for those giving mixed or negative feedback were: *M* = 5.8, *SD* = 3.2, (*t*(26) = 3.160, *p* < .01). Respective credibility ratings were positive: *M* = 7.3, *SD* = 1.8; mixed or negative: *M* = 3.1, *SD* = 2.5, (*t*(26) = 5.307, *p* < .01).

### Qualitative analyses

Themes were grouped according to whether they were MANTRA-specific, SSCM-specific, or non- specific, as well as if they were positive or negative to highlight aspects of the treatment that participants liked or did not like. For MANTRA, specific treatment features were use of the manual and exercises and the focus on cognitive and emotional aspects of AN. For SSCM, treatment-specific factors included the focus on weight and eating and lack of structure. Treatment outcomes and recovery describe the positive and negative outcomes, and stage of recovery at the end of treatment. Non-specific factors describe those shared between the two treatment groups such as duration and frequency of treatment, external support and the patient-therapist relationship. Several similar themes were identified in Lose et al.’s [19] paper and the same theme names have been used here for consistency. The resulting themes and sample quotations are presented in Table 3, and the thematic map in Fig. 1.

**Table 3:** Themes and sample quotations based on written feedback

MANTRA	
Themes and Subthemes	Quotes
Treatment aspects	
1.Use of manual	
* Positive (n = 10)*	M3 “Many of the exercises that we went through in the sessions made me really think about why I felt and acted the way I did and what I really want from life.”
* Negative (n = 2)*	M23 “I found the workbook didn’t help me as I wasn’t learning anything new.”
2.Treatment focus	
* Positive (n = 4)*	M19 “Not too focused on solely the eating disorder per se, but on the many different factors/difficulties that encourage and maintain the eating problems (and other difficulties).”
* Negative (n = 2)*	M43 “Did not find it that helpful. I did not feel that [the therapist] dealt with either the root of the problem, why I lack confidence, or how to deal with it.”
Treatment outcomes and recovery	
1.Positive outcomes	
* Effect on understanding AN symptoms and their impact on behavior (n = 8)*	M16 “[The treatment] was great in helping me to see it as a disease with symptoms as opposed to something wrong with me as a person.”
	M32 “I feel that the therapy has given me a much greater understanding of why I keep this illness and what the illness gives me. I strongly believe that I needed this insight to be able to work on recovery.”
* Effect on feelings and thought processes (n = 13)*	M7 “I found it beneficial as I was able to start to question the [anorexic] voices and thoughts I had regarding food – whereas before I was unable to challenge them and simply accepted them as fact.”
	M41 “By no means have I completely let go of anorexia but I’ve taken so many more steps than I felt capable of before. I have reappraised myself, my eating, and my illness and feel more hopeful than I have in years.”
* Effect on eating habits and weight (n = 7)*	M3 “The food plans we made also helped me to learn how to eat normally and I now rarely worry about what I eat.”
	M33 “I think strategies… help me to manage my anxiousness around food and have increased my self-management of my food intake.”
* Effect on communication (n = 3)*	M1 “[Expressing my feelings to others] has been really beneficial as I can tell people when I am struggling, instead of letting the eating disorder do the talking.”
2.Negative outcomes (n = 1)	M24 “I do not feel [the therapy] has necessarily AIDED recovery.”
3.Stage of recovery (n = 9)	M25 “I still have my fears about gaining weight, weighing myself and find it very difficult to change. Maybe I was/am expecting too much and there is no magic/instant cure or solution to my problems with ED.”
	M42 “I consider myself to be almost completely recovered at this point and very excited about moving forward.”
SSCM	
Treatment aspects	
1.Lack of structure	
* Positive (n = 1)*	S34 “I was pleased to have been given the freedom and time to find a lot of answers and strength for myself, but having the security of monitoring and treatment behind me.”
* Negative (n = 2)*	S23 “I did not feel like there was a secure plan in place for that there were set goals or targets, which I would have found helpful.”
	S33 “Didn’t like the unstructured approach of SSCM, would have preferred more tailored one like MANTRA to ensure lots of aspects of anorexia… were covered.”
2.Focus on Nutrition	
* Positive (n = 1)*	S9 “We talked a lot about food and meal plans and provided me with all the material to work out what I needed to eat and open my eyes to what healthy eating and nutrients really mean.”
* Negative (n = 4)*	S11 “The fact that I got weighed each week terrified me… [The therapist] started talking about 2500 calorie diet, this is when I said no I can’t do it.”
	S31 “I also found the therapy too focused on food (encouragement to eat) as opposed to exploring feeling and behaviors. I would have liked to explore why I do this to myself. I am still struggling to understand this.”
Treatment outcomes and recovery	
1.Positive outcomes	
* Effect on understanding AN symptoms and their impact on behavior (n = 4)*	S7 “I do feel that the therapy has given me a better foundation and understanding of my behavior to work from.”
	S31 “During the therapy I became aware of my issues and realized that most of my physical problems are due to the same issue.”
* Effect on feelings and thought processes (n = 1)*	S26 “I hope it has set me up with the right mental tools to be able to analyze situations better (both related and unrelated to food).”
* Effect on eating habits and weight (n = 2)*	S27 “I still have a lot of negative eating disordered thoughts but I’m at a healthy weight so I suppose that is a positive.”
* Effect on communication (n = 2)*	S30 “Felt that I could be very open and honest even when I knew what I was feeling or saying would expose my underlying desire to keep my eating disorder.”
2.Negative outcomes (*n* = 2)	S1 “Anything new I have tried I have not tried more than once or made a permanent part of my diet… And sometimes I feel I say or agree to things just to please my therapist.”
	S33 “Ended up being admitted as an inpatient due to low mood and then daycare treatment for [eating disorders].”
3.Stage of recovery (*n* = 4)	S17 “I felt like the therapy did not get very far… I don’t know if any therapy can ever stop [the illness].”
	S26 “I hope with carrying on the therapy for a bit I will be able to return to eating healthily.”
Non-specific	
1.Duration, frequency and disruptions of therapy (*n* = 11)	M12 “I do not feel that it was long enough and I have definitely regressed.”
	S14 “I found it difficult to maintain changes to my diet only seeing the therapist once a week, perhaps more regular appointments would help people continue their recovery independently.”
	S12 “Unfortunately, my therapist was away for quite a few weeks during my weekly sessions and this did set me back a bit, and feel I still have quite a way to go.”
2.External social support (*n* = 9)	S31 “It was very useful to have my family involved in the therapy.”
	S21 “I would have liked to have group sessions so I didn’t feel so abnormal about thoughts I was having.”
3.Pacing and individualization of treatment (*n* = 8)	M27 “I appreciated the way it was individualized and went at the pace that suited me.”
	S11 “I think a slower pace, I was not at all ready mentally to increase food.”
4.Therapist (*n* = 31)	M7 “My psychologist was fantastic with me. I feel I learned a lot from her and even though I have now stopped seeing her, I still remember a lot of what we covered and can use it to continue challenging my thoughts.”
	S7 “After developing a therapist/patient relationship… I was able to feel as though I could trust her advice over my thoughts.”
	M48 “The therapist I was given I believe failed to deliver this treatment in an acceptable and appropriate manner.”
5.Concerns about ending therapy (*n* = 9)	M44 “I notice an immediate change back to some old behaviors within two weeks of ceasing weekly sessions.”
	S27 “I am scared of getting ill again.”

*Note.* MANTRA: The Maudsley Model of Anorexia Nervosa Treatment for Adults, SSCM: Specialist Supportive Clinical Management

## MANTRA

### Treatment aspects

#### Use of manual

##### Positive

Ten participants gave positive comments about the MANTRA manual and its exercises. They remarked on its flexibility and ability to be tailored to their needs. Participants also felt that the resources were enjoyable to use, helped them gain better understanding of their illness and provided them with skills to facilitate change. They valued the focused tasks and useful analogies in aiding their reflection and recovery. Even though two participants found the exercises “repetitive” (M20) or “uncomfortable to do” (M16), they indicated that they understood the importance of the issues addressed.

##### Negative

Two participants did not find the manual useful, as they felt that the exercises were too difficult, or that they did not learn anything new from the resources.

#### Treatment focus

##### Positive

Four participants felt that MANTRA’s focus on cognitive, emotional and interpersonal factors, instead of primarily nutrition, helped them better understand their illness.

##### Negative

In contrast, one participant reported that MANTRA did not address the issue of self-confidence which she felt was key for her illness and another would have preferred more help with nutrition.

### Treatment outcomes and recovery

#### Positive outcomes

##### Effect on understanding ED symptoms and their impact on behaviour

Eight participants said that they had benefited from acquiring a greater understanding of AN and of themselves, including the factors contributing to AN, symptoms and their negative impact on their life. Participants were able to see AN as an illness with symptoms separate from their identity. Reflection also helped them to identify, face, and tackle issues from their past and present. Participants highlighted that these factors helped them in their recovery.

##### Effect on feelings and thought processes

Thirteen participants also referred to ways in which the treatment contributed to altered ways of thinking, reappraising their approach to situations in a more adaptive way. This enabled them to challenge negative thoughts, reduce anxiety, accept weight gain, and gain confidence in recovery.

##### Effect on eating habits and weight

Seven participants described positive changes in their eating and weight and linked this to other improvements in their life, such as resuming their education and boosting their self-confidence.

##### Effect on communication

Three participants indicated greater expression of feelings towards others around them, enabling them to open up about their illness to close others.

#### Negative outcomes

One participant commented that the treatment did not help with her recovery but did not elaborate as to why this was the case.

#### Stage of recovery

Several MANTRA participants commented on their stage of recovery. Two conveyed that they still had difficulties with eating, weight gain, and relationships with others. Even though they acknowledged they had not attained full recovery, seven participants expressed hope and excitement, as well as the recognition that recovery would be a difficult but not insurmountable challenge.

## SSCM

### Treatment aspects

Two distinct features of SSCM were highlighted by patients: focus on weight and eating and its lack of structure.

#### Lack of structure

##### Positive

One participant liked the freedom associated with the treatment, as it enabled her to express herself fully with the guidance of the therapist.

##### Negative

Two participants disliked the lack of treatment structure, and would have preferred a more structured and comprehensive approach.

#### Focus on nutrition

##### Positive

One participant found SSCM’s focus on weight and eating favourable in supporting recovery.

##### Negative

Four participants indicated that the focus on weighing and nutritional advice was distressing or unsuitable for their needs. They stated that a focus on other aspects of AN, such as body image, or its emotional and psychological aspects would have been preferable.

### Treatment outcomes and recovery

#### Positive outcomes

##### Effect on understanding ED symptoms and their impact on behaviour

Four SSCM patients expressed that they had gained greater understanding of AN and of themselves, which helped them identify problems that needed addressing.

##### Effect on feelings and thought processes

One participant indicated that they experienced altered ways of thinking, in terms of an improved ability to analyse situations and to positively compare themselves to others.

##### Effect on eating habits and weight

Two participants described achieving normal eating, weight gain, and a greater understanding and respect for their body.

##### Effect on communication

Two participants found the therapy useful in encouraging expression of feelings towards the therapist and close others.

#### Negative outcomes

Two SSCM patients felt that the treatment did not benefit them as they required an additional inpatient admission.

#### Stage of recovery

Four participants shared their outlook regarding their own recovery. One expressed hopefulness about healthy eating, whilst the other three expressed that the therapy did not enable them to achieve long-lasting changes or that recovery was perceived to be impossible.

### Non-specific factors

#### Duration, frequency and disruptions in treatment

Eight participants (*M* = 5, *S* = 3) commented on the duration of treatment and frequency of therapy sessions, with some (*M* = 1) being satisfied and others (*M* = 4, *S* = 3) wishing for a greater number or frequency of sessions. Three participants (*M* = 1, *S* = 2) were dissatisfied about temporary disruptions in their therapy that they felt had contributed to their condition worsening.

#### External social support

Participants valued the importance of social support during their therapy and recovery. Five (*M* = 4, *S* = 1) individuals felt that more involvement of family members and close others would have been beneficial. Four participants (*M* = 1, *S* = 3) suggested that the involvement of other currently ill or recovered AN patients could serve as friends who truly understood their illness and provide role models for recovery.

#### Pacing and individualisation of treatment

Five participants (*M* = 4, *S* = 1) commented that their treatment was flexible, individualized and relevant for their needs. This included the flexibility of treatment, meeting with a dietician for nutritional advice, and treatment moving at a comfortable pace. Three participants (*M* = 1, *S* = 2), however, felt that their treatment was not individualized enough.

#### Therapist

A total of 31 participants (*M* = 21, *S* = 10) mentioned their therapists in their written feedback; 27 (*M* = 19, *S* = 8) gave positive comments, including favorable qualities, such as being understanding, sympathetic, non-judgmental, kind, considerate to their needs, welcoming, intuitive, compassionate, and empathic. Furthermore, they also commented on the therapists’ expertise, and the development of the therapist-patient relationship. Three participants (*M* = 1, *S* = 2), however, expressed dissatisfaction about a change of therapist and disrupted therapy, and one MANTRA patient felt that their therapist lacked training in conducting the treatment.

#### Concerns about ending therapy

Several participants (*M* = 7, *S* = 2) expressed concerns about ending therapy, including fear of returning to old behaviours and suggested including more follow-up support.

## Discussion

This study is the second stage of a process evaluation integrated into a large scale RCT of two outpatient psychological therapies in adults with AN. This evaluation combined the use of both quantitative and qualitative measures to assess patients’ treatment experiences.

Findings show that at 12 months post-randomisation i.e. at the end of both weekly and follow up treatment sessions, MANTRA patients rate their treatment as significantly more acceptable and credible than patients receiving SSCM. This result is strengthened by the clear agreement between the global valence of the qualitative written feedback and quantitative VAS ratings at 12-month follow-up, in that participants who provided overall positive written feedback also rated their treatment as significantly more acceptable and credible than participants who gave mixed or negative written feedback.

It is also of note that, although overall proportions of MANTRA and SSCM patients who provided some process feedback did not differ, significantly more MANTRA patients gave written feedback when compared to SSCM. Those MANTRA patients who did respond also tended to give a more detailed account of their experiences (as assessed by number of words used). This difference might be explained by the fact that MANTRA aims to reduce AN patients’ emotion avoidance and suppression, and to increase emotional clarity and adaptive emotion regulation, such as emotional expressivity [27]. Arguably willingness to express views about one’s treatment experience is a facet of this. Alternatively, it may be that MANTRA patients are simply more practiced in using writing as a form of expression compared to SSCM patients, given that therapeutic writing exercises are incorporated into this treatment, but not into SSCM.

Overall, themes that arose from the thematic analysis replicate those found in our earlier paper which used qualitative interviews in a subset of patients from one participating centre [19]. Participants highlighted both treatment specific (e.g. MANTRA: manual, exercises, comprehensive focus; SSCM: focus on nutrition, lack of structure) and non-specific factors (e.g. relationship with therapist, treatment pacing, duration, frequency and disruption, external social support and concerns about ending therapy) as important aspects of their treatment. Thus MANTRA and SSCM were experienced somewhat differently in terms of their focus, strengths and challenges, but there were also some overlapping features.

The perceived differences between treatments, both positive and negative, were to an extent, in line with the specific focus and targets of each treatment. MANTRA targets motivational, cognitive, emotional and interpersonal maintaining factors of AN with the aim of effecting nutritional changes via broader cognitive and emotional changes. In contrast, SSCM centrally focuses on improving eating and weight and other topics for discussion arise as chosen by patients, session by session. Thus, in MANTRA many participants valued the flexible, individually tailored manual-based approach and the focus on thoughts and feelings. A small number of patients however, experienced certain therapeutic exercises as unhelpful or even as patronising, and felt the manual-based approach did not address their unique problems or teach them anything new. Whilst these more critical views may be legitimate and appropriate there is a possibility that they may at least in part be linked to AN patients’ detail-focused perfectionist cognitive style, that makes some individuals dismissive of anything that is not a perfect fit. It is important for therapist to be aware of this possibility.

In SSCM, patients who gave positive feedback commended the clear focus on nutrition and freedom offered, whereas others saw the focus on nutrition as negative, generating anxiety and distress. SSCM’s lack of structure was also seen as unhelpful by some, in facilitating exploration of certain aspects of their illness.

In addition, across treatment groups disappointment regarding failure to reach desired improvements or a continuation of significant ED symptoms associated with more negative feedback. Thus mixed or negative feedback about treatment appeared to be due to a range of reasons, some to do with the therapeutic focus, content, delivery or structure, others with patients’ mixed feelings about change or disappointment about limited progress.

As expected, due to feedback being collected at the end of treatment the theme of recovery and life beyond therapy was more prominent in the present evaluation than in our earlier interview study [19]. More MANTRA patients addressed this issue than those receiving SSCM. In general, MANTRA patients expressed greater satisfaction with their progress and excitement about working towards their future, despite the recognition that achieving recovery would remain challenging. Of the few SSCM patients mentioning this theme one individual expressed pessimism about recovery whilst the other remained hopeful.

Several non-specific factors were mentioned by a substantial number of patients. This suggests the importance of basic factors such as structure, duration and timing for the success of a treatment and the difficulties that arise from disruptions through therapist illness or departure. The single most commonly mentioned theme by a long way, unsurprisingly was that of the therapeutic relationship A previous study on therapist characteristics preferred by AN patients identified factors (acceptance, vitality, challenge and expertise) associated with a good therapeutic relationship and also unhelpful therapist characteristics (disregard, prejudice, passivity, pampering) [28]. The views of patients in the present study largely confirm these findings. Overall, our findings support the implications highlighted in the earlier process evaluations of the MOSAIC Trial [19, 20]. More specifically, the importance of non- specific factors, such as therapist characteristics, individualisation of treatment and social support are emphasized.

Since the completion of the process study, the study’s main outcomes have been published [18]. Findings show that MANTRA patients attended significantly more treatment sessions and a higher proportion of MANTRA patients were treatment completers. Whilst overall there were no significant differences at 12 months between MANTRA and SSCM on BMI, eating disorders psychopathology, distress levels and other outcomes, MANTRA appeared to be advantageous in more underweight patients in terms of facilitating greater weight gain at both 6 and 12 months. This suggests that MANTRA patients’ view of this treatment as more acceptable, credible and all round positive is at least in part rooted in better clinical outcomes.

The ‘three-legged stool’ principle of evidence-based medicine [29, 30] suggests that in choosing one intervention over another, best available research evidence about efficacy, clinical expertise and patient preference all need to be considered. With this in mind MANTRA can be recommended over SSCM.

Finally, due to the more positive initial treatment experience, individuals’ receiving MANTRA may be more willing to have further treatment should this become necessary. This is particularly important for the many individuals with AN who have a chronic and/or relapsing course of the illness.

This study has several strengths and limitations. Firstly, over 80 % of MOSAIC trial participants provided some process feedback and there were no baseline differences between those who did and did not provide these data. Thus, our findings are likely to be representative of the views shared by the whole MOSAIC trial sample. Secondly, treatment acceptability and credibility ratings were supported by the written feedback provided. Thirdly, participants were recruited from all treatment sites involved in the MOSAIC trial and data were obtained and analysed before trial outcomes are known. Finally, bias in the interpretation of qualitative data was reduced through performing data triangulations.

The study also has a number of limitations. First, a significantly higher proportion of MANTRA patients compared to SSCM patients provided written feedback. The fact that the SSCM group were more selected may have biased the results. Second, although the use of written feedback, enabled participants to express their opinions freely and independently (i.e. away from their therapists’), the questions asked were broad and the written nature of the feedback did not allow for probing and exploration of key issues as a qualitative interview would. It might have been helpful to include detailed questions in order to obtain more in-depth feedback regarding different treatment components and outcomes. Third, the feedback represents participants’ retrospective recollections of treatment at the time point of assessment, and there may have been some recall bias. However, this would have operated similarly in both treatments. Fourth, some of the qualitative themes were only reported by one or two participants. Thus caution is required in interpretation of these findings. Nonetheless, current results replicate those obtained from semi-structured interviews carried out as part of the previous evaluation [19].

Fifth, our assessment of credibility was limited to helpfulness of treatment and thus rather narrow. Established expectancy/credibility scales [25] also include other aspects, such as how logical a particular therapy rationale seems to the patient and whether they would recommend a treatment to a friend or another person in their situation. It is possible that use of a broader credibility scale would have yielded different responses.

A final limitation is the lack of a treatment expectancy measure taken at the start of therapy. However, in the previous pilot trial comparing MANTRA with SSCM a treatment expectancy measure was included and both treatments were viewed as equally credible and positive [15].

## Conclusions

Together with our earlier interview paper [19] on patients’ views of their treatment this study is very much a first attempt to capture some of the qualitative views of patients receiving the two study treatments and is somewhat exploratory in its nature. MANTRA patients viewed their treatment as more acceptable, credible and generally more positive in comparison to patients receiving SSCM at 12 months. Participants also highlighted the useful components of and outcomes associated with their treatment. MANTRA patients valued the use of the manual and the focus on cognitive, emotional and interpersonal factors, while SSCM patients mentioned its less structured nature and focus on nutrition. This study, together with the previous process evaluation [19], suggests that many individuals with AN perceive MANTRA as a viable treatment option. Further studies should include more sophisticated methods of evaluating treatment process to address the dearth of research in this area.
